# Culturing neutrophils from human CD34^+^ HSPCs: Why medium matters

**DOI:** 10.1016/j.xpro.2026.104844

**Published:** 2026-09-23

**Authors:** Angela A.F. Gankema, Fleur van Oosterom, Taco W. Kuijpers

**Affiliations:** 1 Sanquin Research, University of Amsterdam, Amsterdam 1066 CX, the Netherlands; 2 Department of Pediatric Immunology, Rheumatology and Infectious Diseases, Emma Children’s Hospital, Amsterdam University Medical Center (Amsterdam UMC), University of Amsterdam, Amsterdam 1105 AZ, the Netherlands

**Keywords:** CD34^+^ HSPCs, cell culturing, medium, neutrophils, SL-II, stemMACS

## Abstract

The cell culturing field is constantly improving cell differentiation protocols to better study and recapitulate human immune cells. The most appropriate culturing medium, which growth factors to use, and supplementation of cytokines need to be optimized. In this study, two cell culturing media for differentiating CD34^+^ hematopoietic stem and progenitor cells (HSPCs) into neutrophils were compared. Iscove’s Modified Dulbecco’s Medium (IMDM) resulted in hybrid neutrophil-like cells, expressing higher levels of CD14 and HLA-DR next to the typical neutrophil surface proteins. Serum-free Stemline-II (SL-II) medium resulted in cells closely resembling functional human blood neutrophils. Since CD34^+^ HSPCs cultured in SL-II medium outperform the more standard IMDM in neutrophil differentiation, a viable replacement for the discontinued SL-II medium is required. Lonza TheraPEAK X-*Vivo* 15 Serum-Free Hematopoietic Cell Medium, Cell Genix GMP Stem Cell Growth Medium, and StemMACS PSC-Brew XF were tested as possible replacements, from which StemMACS PSC-Brew XF seems most promising.

## Highlights

• Culturing CD34^+^ stem cells in SL-II medium leads to functional neutrophils
• Culturing CD34^+^ stem cells in IMDM results in a hybrid cell type
• StemMACS seems to be promising replacement for SL-II medium

## Graphical abstract

**Figure:**
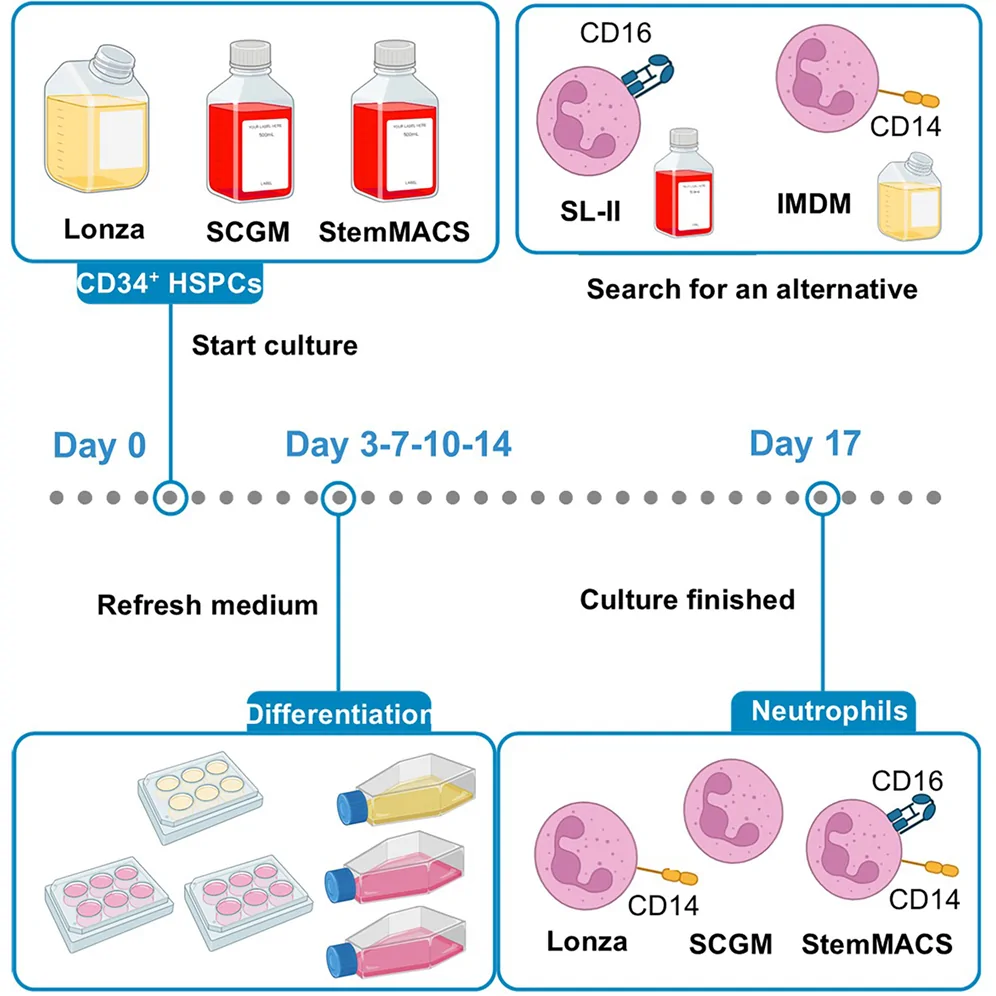

## Teaser

Gankema et al. demonstrate that human CD34^+^ hematopoietic stem and progenitor cells can successfully be differentiated towards neutrophils. However, the choice of medium highly impacts the maturation of cells. The recently discontinued Stemline-II medium showed the most successful results. The authors have tried to look for an alternative medium.

## Introduction

Cell culturing has gained interest in many fields of research, but particularly in the field of hematology and cancer immunotherapy. To reach the high blood demand, culturing blood products could decrease the burden on blood banks.^1^ Furthermore, new cellular therapies combating cancer often rely on specific immune cells, for example T cells or NK cells that can be engineered *ex vivo*.^2^ Culturing these immune cells could well lead to an off-the-shelf therapy, resulting in faster and cheaper treatment of patients.^3^ To culture specific cell types, optimization of culturing conditions is required. Therefore, it is important to find the most optimal culturing medium, cell concentration, cytokines and growth factors, as well as the optimal length for culturing and timepoints to refresh the medium.

Polymorphonuclear neutrophils (PMNs) are the most abundant leukocyte in the blood, comprising 50–70% of all leukocytes.^4^ Upon infection, these cells are the first responders leading to killing of pathogens by release of their toxic granular content or elimination of the pathogen by phagocytosis.^5^ However, studies on molecular mechanisms behind neutrophil functions are still lagging behind. They are impeded by their short lifespan, the easy activation upon cell isolation from the blood, the high donor variation in primary human material, and the poor resemblance of cell lines and murine neutrophils to circulating human neutrophils.^6^^,^^7^^,^^8^^,^^9^^,^^10^ Therefore, it is of interest to culture *in vitro* neutrophils from human CD34^+^ hematopoietic stem and progenitor cells (HSPCs). This does not only facilitate studying neutrophil maturation and function but also creates an opportunity to introduce genetic modifications by CRISPR- Cas9 to mimic certain diseases. This leads to better understanding of neutrophil biology and could ultimately lead to improved treatment of patients with neutrophil defects. Furthermore, in the future, cultured neutrophils might be transfused to patients lacking functional neutrophils and therefore help them clear infections.^11^ Nevertheless, a well-established and validated cell culturing system leading to fully mature functional neutrophils was lacking. Earlier established stem cell-derived neutrophil protocols resulted in neutrophil-like cells but generally fell short to recapitulate terminally differentiated neutrophils.^12^^,^^13^^,^^14^^,^^15^^,^^16^

In our latest study, we compared two different culturing media to generate neutrophils from CD34^+^ HSPCs, the current golden standard in the field of stem cell culturing protocols Iscove’s Modified Dulbecco Medium (IMDM) and the less known Stemline II (SL-II) medium.^17^ Both media were supplemented with the same growth and differentiation factors. After 17 days of culturing, we obtained neutrophil-like cells characterized by CD15^+^CD11b^+^CD66b^+^ expression using both culturing methods with a higher CD16^high^ cell yield for cells cultured in SL-II medium. Further phenotypic and functional analysis showed that neutrophils obtained from the SL-II culture resembled peripheral blood neutrophils better than those obtained from the cells cultured in IMDM medium.^17^ Full characterization of the neutrophils cultured in two different media was performed using both transcriptomics and proteomics, highlighting the differences due to medium choices.^17^ Taken together, we aim to stress the importance of choosing the optimal culture medium for differentiation of CD34^+^ HSPCs to neutrophils using our protocol. Particularly, in the case of neutrophil differentiation, the choice of SL-II medium over IMDM leads to phenotypically and functionally differentiated neutrophils.

Here, we describe a detailed protocol for culturing CD34^+^ HSPCs towards neutrophils in SL-II medium. However, since the production of SL-II has been discontinued, a replacement for SL-II is required. While the manufacturer may consider to restart production since SL-II seems to be an ideal medium with full neutrophil effector functionality while devoid of the unwanted immunosuppressive (side) effect, we tested three different culturing media often mentioned in HSPC culturing protocols to replace SL-II, which are Lonza TheraPEAK X-Vivo 15 Serum-Free Hematopoietic Cell Medium (Lonza), Cell Genix GMP Stem Cell Growth Medium (SCGM), and StemMACS PSC-Brew XF (StemMACS) to test for yield and neutrophil differentiation. While SCGM failed to induce differentiation towards neutrophils, both Lonza and StemMACS media showed expression of typical neutrophil surface proteins on day 17 of the culture. However, just like IMDM, Lonza medium failed to induce high CD16 expression and showed upregulation of CD14 and HLA-DR, suggesting a more hybrid phenotype. StemMACS-derived neutrophils more closely resembled SL-II-cultured neutrophil surface protein expression and function. Therefore, if there will be no industrial solution for the continuation of SL-II production, we suggest optimization of StemMACS for culturing CD34^+^ HSPCs towards neutrophils may form an alternative and have listed potential factors to be optimized in the discussion.

## Results

### Differentiation of human CD34^+^ HSPCs into neutrophils can be performed in SL-II and IMDM

In this study, we applied our previously described 17-days culturing protocol for differentiation of CD34^+^ HSPCs to neutrophils.^17^ During these 17 days, cells are cultured in IMDM or SL-II medium supplemented with growth and differentiation factors (Figure 1A). The medium is refreshed every 3–4 days. Final assessment of neutrophil differentiation was performed on day 17 of the culture. Samples were collected for morphological and phenotypical assessment as well as tested for the ability of neutrophils to produce reactive oxygen species (ROS).

**Figure 1:**
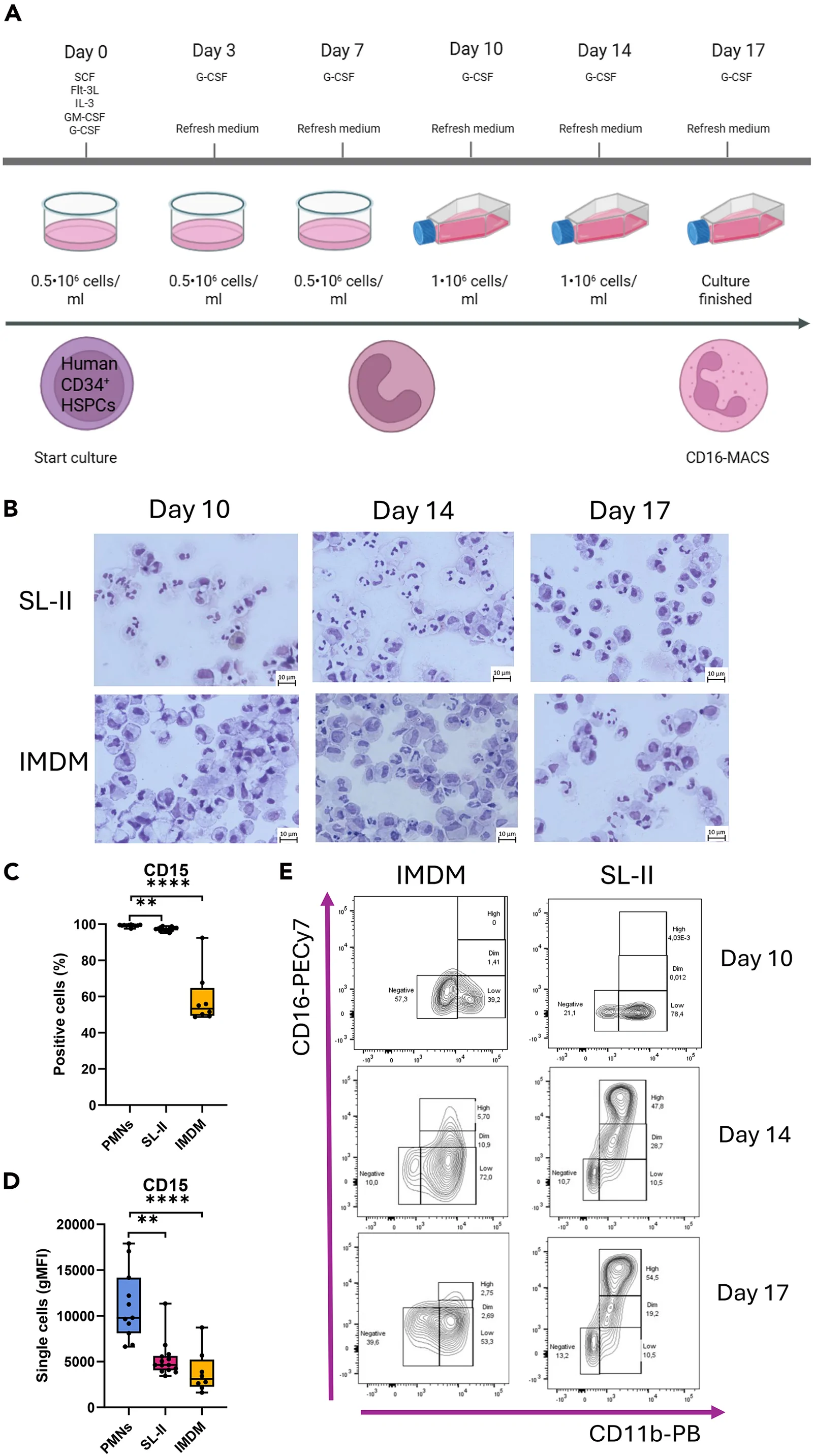
Human CD34^+^ HSPC-derived neutrophils cultured in either IMDM or SL-II medium (bulk) (A) Culturing method starting from human CD34^+^ HSPCs towards neutrophils in 17 days. (B) Representative cytospins of bulk CD34^+^ HSPC-derived neutrophils cultured in IMDM or SL-II medium for 10, 14 or 17 days after Giemsa-May staining. Scalebars set at 10 μm. n=8–14. (C) Percentage of CD15^+^ neutrophils on day 17 of the culture compared to PMNs gated on the cell population followed by single cells. n=8–14. (D) Geometric Mean Fluorescent Intensity (gMFI) of CD15 expression of bulk neutrophils gated on the cell population followed by single cells on day 17 of the culture compared to PMNs. n=8–14. (E) Representative histograms of CD11b/CD16 expression on CD34^+^ HSPC-derived neutrophils cultured in IMDM vs SL-II medium for 10, 14 or 17 days measured by flow cytometry. Gated on the cell population followed by single cells and CD15^+^ cells. n=8–14. ∗∗ *p* <0.01, ∗∗∗∗ *p* <0.0001. Data are represented as median and interquartile range. Each datapoint represents an independent donor and the graphs show pooled data of different donors. Normality was tested with a Shapiro-Wilk test. P values were calculated using a Kruskal-Wallis followed by Dunn’s post hoc multiple comparison test.

First, comparing cellular morphology, both CD34^+^ HSPCs cultured in IMDM and in SL-II medium showed an increase in cells with the typical neutrophil multi-lobular nucleus during different days of the culture by May-Grünwald and Giemsa staining (Figure 1B). Based on flow cytometry data on CD15 expression, both CD34^+^ HSPC-derived neutrophils cultured in IMDM and SL-II in bulk show CD15^+^ cells on day 17. While the percentage of CD15^+^ cells cultured in SL-II is higher and more in line with PMNs, the geometric mean fluorescent intensity is comparable between IMDM and SL-II (Figures 1C and 1D). Gating on CD15^+^ cells, the percentage of CD11b^+^CD16^+^ cells was determined. Both cultures show an increase in CD11b^+^ cells over time, while more mature CD16^+^ cells were only found in the SL-II cultured neutrophils from day 14 of the culture onwards (Figure 1E).

### Culturing CD34^+^ HSPCs in SL-II or IMDM leads to different maturation processes

While both media led to neutrophil-like cells based on typical neutrophil markers such as CD66b and SIGLEC-9, the percentages of positive cells were lower for CD34^+^ HSPC-derived neutrophils cultured in IMDM (Figures 2A and 2B). In addition, CD14, a marker normally weakly expressed on neutrophils, is increased in percentage on IMDM cultured neutrophils versus neutrophils cultured in SL-II or PMNs (Figure 2C). Similar results could be found for CD29, a marker for β1 integrin expression, showing increased percentages for IMDM cultured neutrophils. Moreover, HLA-DR was also found on IMDM cultured neutrophils while absent on PMNs or SL-II cultured neutrophils. In contrast, peripheral blood neutrophils typically express low to moderate levels of CD29 and lack HLA-DR expression. Therefore, the expression levels of these markers on IMDM cultured neutrophils suggest a more hybrid phenotype.

**Figure 2:**
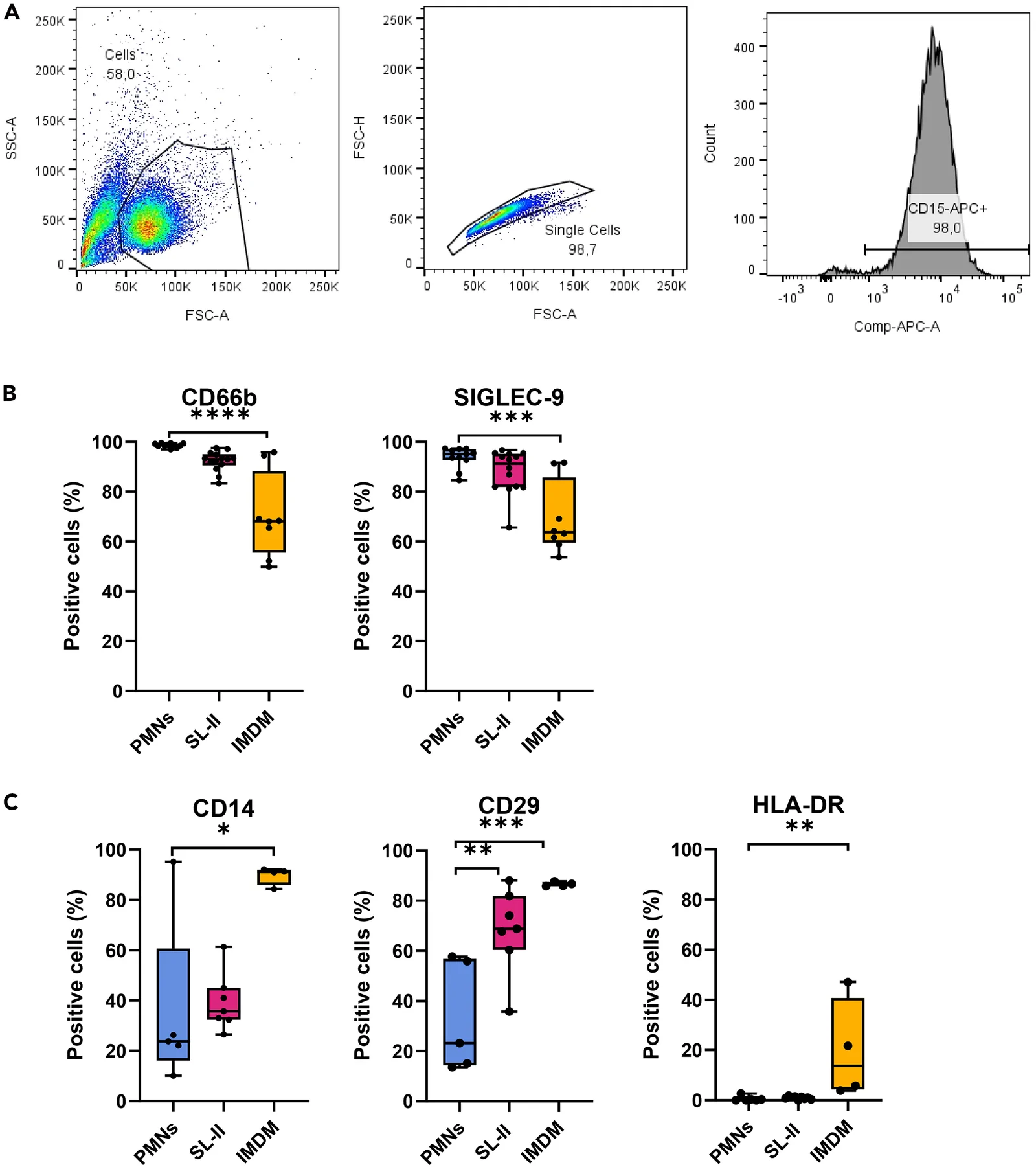
Culturing medium affects protein expression in CD34^+^ HSPC-derived neutrophils (A) Gating strategy for flow cytometry. (B) The percentage of cells expressing neutrophil-specific markers such as CD66b and SIGLEC-9 were similar between CD34^+^ HSPC-derived neutrophils cultured in SL-II (pink) compared to PMNs (blue) while CD34^+^ HSPC-derived neutrophils cultured in IMDM (orange) were lower. Gated on the cell population followed by single cells and CD15^+^ cells. n=8–14. (C) Differences in the percentage of cells expressing surface markers CD14, CD29, and HLA-DR between CD34^+^ HSPC-derived neutrophils cultured in SL-II (pink) or IMDM (orange) compared to PMNs (blue). Gated on the cell population followed by single cells and CD15^+^ cells. n=4–7. ∗*p* <0.05, ∗∗ *p* <0.01, ∗∗∗ *p* <0.001, ∗∗∗∗ *p* <0.0001. Data are represented as median and interquartile range. Each datapoint represents an independent donor and the graphs show pooled data of different donors. Normality was tested with a Shapiro-Wilk test. P values were calculated using a Kruskal-Wallis followed by Dunn’s post hoc multiple comparison test or an ordinary one-way ANOVA followed by Dunnett’s post hoc multiple comparison test.

These data show that neutrophils cultured from CD34^+^ HSPCs in SL-II medium are phenotypically more like PMNs compared to cells cultured in IMDM, especially when looking at CD16^+^ expression over time. Since CD16 is a neutrophil marker that specifically comes up at later stages during differentiation, the low expression levels in the IMDM cultures suggest that these cultured neutrophils do not reach full maturation or differentiation, while they do in SL-II medium. Therefore, MACS sorting for CD16 positive cells to collect the mature population of neutrophils was limited to the SL-II cultured neutrophils. These CD16^+^ neutrophils showed a high similarity to circulating neutrophils (Webbers et al., 2025).

### Alternatives for SL-II medium show limited differentiation towards neutrophils

Recently, it has been brought to our attention that production of SL-II medium is discontinued at Merck. Therefore, testing alternative culturing media leading to similar neutrophil phenotype and functionality as SL-II medium is of great importance. For that reason, a literature search was performed for alternative media used in the field of CD34^+^ HSPC culturing. So far, we tested three different stem cell media: Lonza TheraPEAK X-Vivo 15 Serum-Free Hematopoietic Cell Medium (Lonza), Cell Genix GMP Stem Cell Growth Medium (SCGM), and StemMACS PSC-Brew XF (StemMACS). Lonza medium has been described by multiple studies for the maintenance of hematopoietic stem cells,^18^^,^^19^ as well as for differentiation of monocytes from PBMCs and iPSCs into macrophages.^20^^,^^21^ SCGM has been used for the expansion of human CD34^+^ cells from cord blood.^22^ StemMACS is designed for efficient maintenance and expansion of human pluripotent stem cells.^23^ However, studies looking into differentiation of CD34^+^ HSPCs into neutrophils with these media have not been published yet.

First of all, CD34^+^ HSPCs were cultured in different media, and cells were counted every 3–4 days during medium refreshment to determine the cumulative cell growth (Figure 3A). CD34^+^ HSPCs cultured in SCGM did not proliferate. Both CD34^+^ HSPCs in Lonza and in StemMACS medium proliferated but the yield was lower compared to SL-II medium. On day 10, 14 and 17, morphology of the cells was assessed by imaging cytospins (Figure 3B). During cell culturing the nucleus of the CD34^+^ HSPC becomes multilobular, a characteristic of neutrophils, however the multilobular nucleus was not observed in SCGM and a few cells in Lonza showed this characteristic. StemMACS showed most neutrophils with a segmented nucleus starting from day 14 onwards to day 17 of the culture. These results are in line with the CD66b and SIGLEC-9 positive cells that are most comparable to neutrophils in SL-II medium and PMNs for stemMACS cultured neutrophils (Figure 4A). Although culturing CD34^+^ HSPCs in all three media does lead to neutrophils to some extent, CD16 expression is low in both Lonza and SCGM showing more comparable expression to the IMDM cultured neutrophils (Figure 3C**)**. Moreover, SCGM media did not lead to satisfactory expansion of cells, leading to a very low yield (Figure 3A**)**. StemMACS more closely resembled the SL-II derived neutrophils in yield, morphology, maturation, and surface marker expression (Figures 3A–3C and 4A).

**Figure 3:**
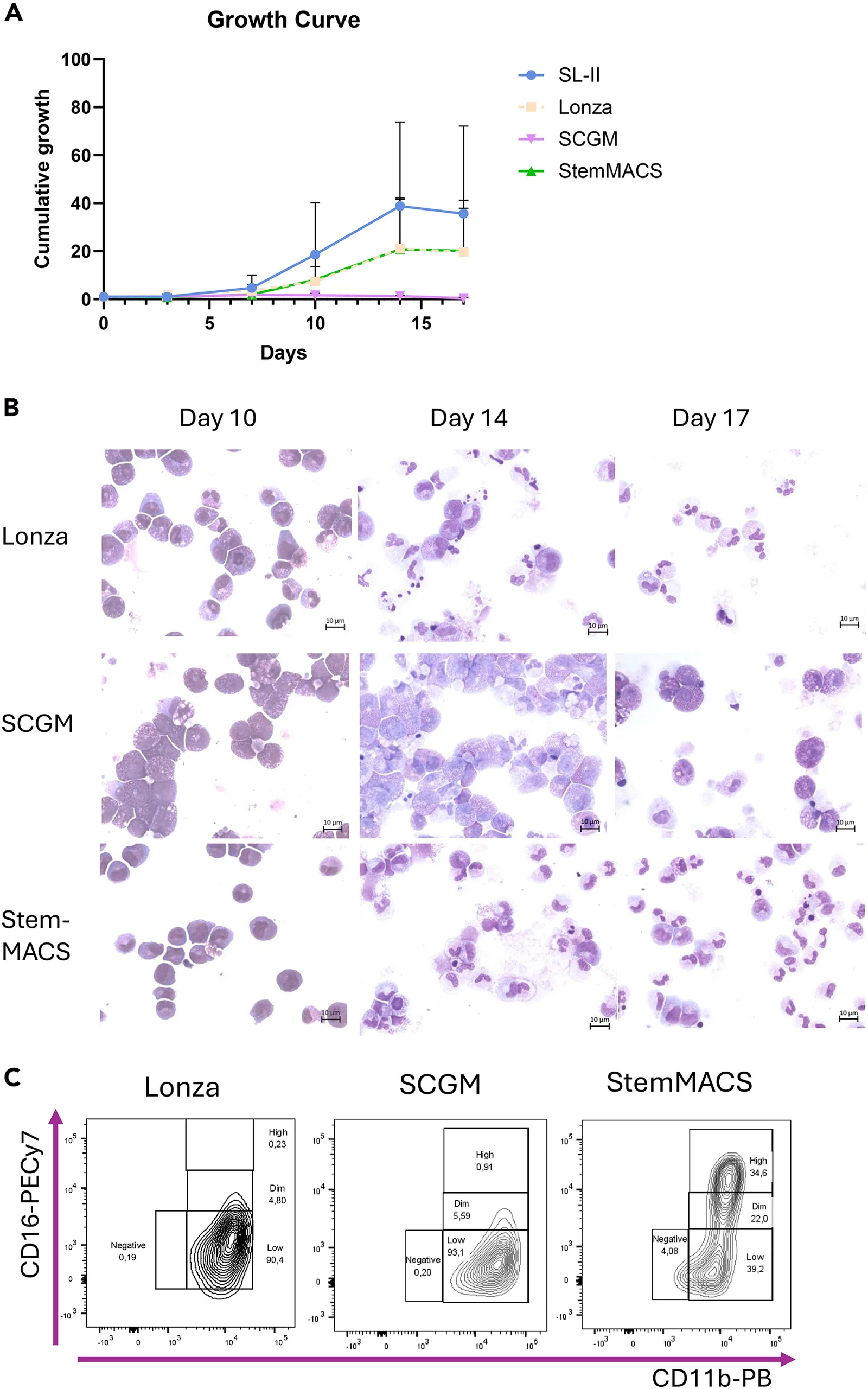
A replacement for SL-II medium leading to mature CD34^+^ HSPC-derived neutrophils is tested (A) Cumulative growth curve for CD34^+^ HSPC-derived neutrophils cultured in SL-II, Lonza, SCGM, or StemMACS medium during 17 days. n=1–5. Mean values are shown and error bars indicate the standard deviations. (B) Representative cytospins of CD34^+^ HSPC-derived neutrophils in different media (Lonza, SCGM, StemMACS) on day 10, day 14 and day 17 of the culture. Scalebars set at 10 μm. n=1–4. (C) Representative flow cytometry plots of CD11b/CD16 expression to assess maturation of the CD34^+^ HSPC-derived neutrophils on day 17 of the culture. Gated on the cell population followed by single cells and CD15^+^ cells. n=1–4.

**Figure 4:**
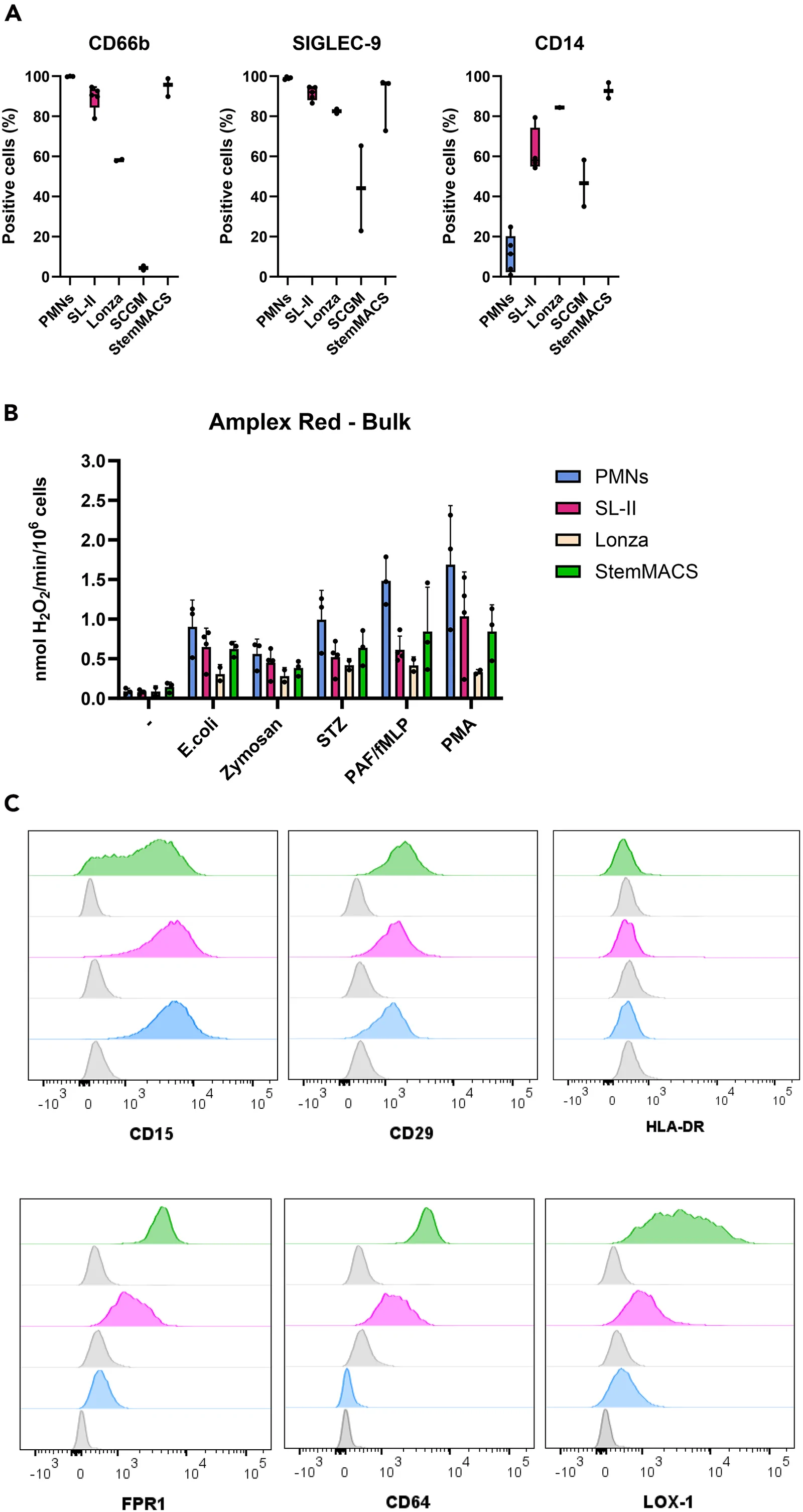
Alternatives for SL-II medium show differential surface marker expression (A) Comparing the percentage of different surface markers on CD34^+^ HSPC-derived neutrophils cultured in SL-II, Lonza, SCGM, or StemMACS medium to PMNs. Data are represented as median and interquartile range. Gated on the cell population followed by single cells and CD15^+^ cells. n=2–5. (B) Extracellular ROS production (bulk) to assess functionality of the CD34^+^ HSPC-derived neutrophils cultured in SL-II (pink), Lonza (beige) or StemMACS (green) compared to PMNs (blue). Error bars indicate the standard deviation. n=2–3. (C) The expression levels of different neutrophil (activation) markers like FPR1, CD64 and LOX-1 on CD34^+^ HSPC-derived neutrophils cultured in SL-II (pink), Lonza (beige) or StemMACS (green) compared to PMNs (blue). Representative histograms are shown. Gated on the cell population followed by single cells and CD15^+^ cells. n=4–5.

### Room for improvement for replacing SL-II medium

Assessment of one of the most important neutrophils functions, the production of reactive oxygen species (ROS), shows a similar result for SL-II and StemMACS, both closely recapitulating circulating neutrophils (Figure 4B). Since both SL-II and StemMACS show bigger populations of CD11b^+^CD16^high^ neutrophils, we MACS sorted for these most mature cells which led to more cells with a multi-lobular nucleus. Comparing ROS production between PMNs, CD16^+^ SL-II derived neutrophils, and CD16^+^ StemMACS derived neutrophils shows an increased response to most stimuli for StemMACS derived neutrophils (**data not shown).** This heightened response could indicate that these cells might have more receptors for the tested stimuli or that these cells are slightly pre-activated. Surface marker expression of CD15, CD29 and HLA-DR is comparable between SL-II and StemMACS. Looking at surface marker expression of activating receptors, including FPR1, CD64, and LOX-1 supports the hypothesis for pre-activation (Figure 4C**)**. FPR1 and CD64 are known to be upregulated upon stimulation.^24^^,^^25^ LOX-1 is stored in granules of neutrophils and is detected on the cell surface upon degranulation.^26^ While StemMACS seems to be a promising replacement for SL-II, optimization and further testing will be required to enhance yield and obtain functional neutrophils for proper use *in vivo*.

## Discussion

In this study, we have described a detailed protocol for the differentiation of human CD34^+^ HSPCs towards neutrophils using different cell culturing media. Where the golden standard medium IMDM failed to recapitulate mature PMNs, culturing in SL-II resulted in mature and functional neutrophils, only slightly differing from circulating PMNs. However, since the discontinuation of SL-II medium by Merck was announced, an alternative has to be found. We tested three different stem cell media for their ability to produce mature neutrophils from CD34^+^ HSPCs. Both Cell Genix GMP Stem Cell Growth Medium and Lonza TheraPEAK X-Vivo 15 Serum-Free Hematopoietic Cell Medium did not lead to mature CD16^+^ neutrophils, and Cell Genix GMP Stem Cell Growth Medium also led to a very low cell yield. However, StemMACS PSC-Brew XF did lead to a high percentage of CD16^+^ cells that phenotypically and functionally resemble SL-II neutrophils and PMNs.

While we have defined the optimal conditions for differentiation of CD34^+^ HSPCs towards neutrophils in SL-II medium, the optimal conditions in StemMACS might be different. To increase cell yield, higher concentrations of G-CSF could be considered, since G-CSF is known to promote neutrophil survival and maturation.^27^ Furthermore, c-kit ligand has been shown to enhance HSPC survival, whereas G-CSF had no effect.^28^ Therefore, adding c-kit ligand might improve cell numbers as well. However, c-kit ligand does not induce more cell proliferation but only improves cell survival.

Another option would be to tweak the medium by supplementing amino acids. The amino acid composition of culturing medium is designed to enhance cell growth and productivity. However, amino acid compositions in different culture media vary. The exact composition is often unpublished and therefore unknown. Specific cells might have specific needs in order to mature and carry out their functions. Glutamine has been directly linked to neutrophil cell survival.^29^ Neutrophils require more glutamine than other leukocytes to carry out their basic functions, including ROS production and mitochondrial functionality.^30^ Although neutrophils are heavily dependent on glycolysis and do not rely on oxidative phosphorylation per se, mitochondria play a vital role in redox signaling and apoptosis.^31^^,^^32^ For this latter process, mitochondria of neutrophils are known to contain cytotoxic proteins that can be released upon neutrophil apoptosis to activate caspases.^32^ Furthermore, mitochondria can act as sensors in redox signaling as well as being scavengers for free Ca^2+^, leading to a balance between pro- and antiapoptotic proteins, ultimately determining neutrophil cell survival.^32^ Therefore, mitochondrial functionality is often linked to cell death.^33^ For that reason, adding glutamine to the culture medium might improve cell survival and function. Both taurine and leucine have also been reported to prolong neutrophil cell survival.^34^ Titrating these different amino acids into the medium of choice could improve cell yield.

Additionally, neutrophils derived from the StemMACS culture showed signs of prior activation. Literature shows that G-CSF stimulation of neutrophils leads to stabilization of the CD64 mRNA and upregulates expression.^24^ Similarly, FPR1 mRNA may get stabilized after stimulation with different neutrophil stimuli.^25^ Another sign of activation is the elevated level of CD14 on StemMACS-derived neutrophils. CD14 is stored in azurophilic granules. Activation leads to degranulation which brings CD14 to the surface of neutrophils.^35^ Whether the amount of G-CSF added to the medium induces activation in StemMACS cells and should be altered, remains unclear to date. In a study comparing G-CSF and GM-CSF stimulation, neutrophils showed higher levels of lactoferrin and chemotaxis upon G-CSF stimulation compared to GM-CSF stimulation.^36^

While the obtained results show a clear pattern of how the different tested media affect cell differentiation, only a limited number of healthy CD34^+^ HSPC donors were tested and these results also showed donor dependence. Only three different culturing media were tested for the replacement of SL-II, while the market for HSPC cell culturing medium includes additional media to be considered. In this study, we want to underscore the research implications of the sudden discontinued production of a specified culture medium, and indicate that the choice of medium highly influences yield and cell differentiation.

### Limitations of the study

A limitation of the study is that differences in sex and age of the CD34^+^ HSPC donors on neutrophil outcome are not studied.

## Resource availability

### Lead contact

Further information and requests for resources and reagents should be directed to and will be fulfilled by the lead contact, Angela Adriana Francisca Gankema (a.a.f.gankema1@amsterdamumc.nl).

### Materials availability

This study did not generate new unique reagents. All materials used in this study are commercially available or can be obtained from the sources listed in the key resources table.

### Data and code availability

This study did not generate or analyze original code.

## Acknowledgments

We would like to acknowledge Steven Webbers for his work on the initial culturing experiments in comparing SL-II and IMDM cultured neutrophils. Furthermore, we would like to thank Helin Tercan and Felipe Rojas-Rodriguez for providing feedback on the initial version of the manuscript. This research is funded by Stichting Universitas.

## Author contributions

A.A.F.G. and F.v.O. wrote the STAR report-type article and performed the experiments. T.W.K. provided supervision and feedback.

## Declaration of interests

The authors declare no competing interests.

## STAR★Methods

### Key resources table

**Table:** 

REAGENT or RESOURCE	SOURCE	IDENTIFIER
**Antibodies**		

Pacific Blue™ Mouse Anti-Human CD11b (Clone ICRF44) (1:200)	BD Biosciences	Cat #558123; RRID:AB_397043
CD11b APC (Clone D12) (1:200)	BD Biosciences	Cat #333143; RRID:AB_2868644
PE-Cy™7 Mouse Anti-Human CD14 (Clone M5E2) (1:200)	BD Biosciences	Cat #557742; RRID:AB_396848
CD15 FITC (Clone MMA) (1:150)	BD Biosciences	Cat#332778; RRID:AB_2868627
PE-Cy™7 Mouse Anti-Human CD16 (Clone 3G8) (1:1000)	BD Biosciences	Cat #557744; RRID:AB_396850
Mouse anti Human CD64 antibody (Clone 10.1) (1:100)	Bio-Rad	Cat #MCA756F; RRID:AB_321799
Mouse Anti-Human CD66b Monoclonal antibody, FITC Conjugated (Clone 80H3) (1:50)	Bio-Rad	Cat #MCA216F; RRID:AB_2077860
Human FPR1 Fluorescein-conjugated Antibody (Clone 350418) (1:100)	R&D Systems	Cat #FAB3744F; RRID:AB_2105317
FITC Mouse Anti-Human HLA-DR (Clone G46-6) (1:200)	BD Biosciences	Cat #555811; RRID:AB_396145
PE anti-human LOX-1 Antibody (Clone 15C4) (1:100)	BioLegend	Cat #358603; RRID:AB_2562180
PE anti-human Siglec-9 Antibody (Clone K8) (1:100)	BioLegend	Cat#351503; RRID:AB_10722615

**Bacterial and virus strains**		

*Escherichia coli*	N/A	Strain ML35

**Chemicals, peptides, and recombinant proteins**		

Fetal Calf Serum (FCS)	Bodinco	Cat#BDC-16975; Lot#20120217
FMS-like tyrosine kinase 3 ligand (Flt-3L)	Stem Cell technologies	Cat#78137
Granulocyte-macrophage colony stimulating factor (GM-CSF)	PeProTech	Cat#300–03
Granulocyte colony-stimulating factor (G-CSF)	Neupogen, clinical grade	N/A
Interleukin 3 (IL-3)	Stem Cell Technologies	Cat#78194
N-formyl-methionyl-leucyl-phenylalanine (fMLP)	Sigma-Aldrich	Cat#F3506
Human Stem cell growth factor (hSCF)	Produced in house (Sanquin)	N/A
Phorbol 12-myristate 13-acetate (PMA)	Sigma-Aldrich	Cat#P8139
Platelet Activation Factor (PAF)	Sigma-Aldrich	Cat#P4904
Zymosan	Santa Cruz Biotechnology	Cat#sc-258367

**Software and algorithms**		

FlowJo 10	FlowJo LCC	https://www.flowjo.com
Graphpad	Prism	https://www.graphpad.com

**Other**		

CANTO II	BD	N/A
InfinitePro2000 platereader	Tecan	N/A

### Experimental model and study participant details

#### Human blood donors

Peripheral blood or mobilized peripheral was collected from healthy donors in accordance with Dutch regulations following the Declaration of Helsinki. Donors provided written informed consent approved by Sanquin internal Ethical Advisory Board (Privacy Declaration 1909–0176). All donors were aged between 18 and 65 years, with an equal distribution of males and females. As described as a limitation of the study we did not perform sex- and age related analyses. The number of donors tested can be found in the figure legends.

### Method details

**Timing: 3 h**

**Timing: 1 to 2 h**

**Timing: 17 days, 1 h to refresh medium on day 3, 7, 10, and 14**

#### Culturing protocol

The protocol below describes the specific steps for culturing neutrophils from CD34^+^ HSPCs in Stemline II (SL-II) medium, Iscove’s Modified Dulbecco Medium (IMDM) or one of the other proposed replacement media. The protocol for full differentiation towards neutrophils takes 17 days in total. Assessment of differentiation takes place on day 10, 14 and 17 of the culture.

##### Isolation of CD34^+^ HSPCs

1. Prepare MACS buffer.
  a. Take 500 mL sterile phosphate-buffered solution (PBS) and add 25 mL of filter-sterile 10% BSA and 2 mL of 0.5 M EDTA.
    i. For 0.5 M EDTA stock solution 9.3gr di-sodium-ethylenediaminetetraacetate dihydrate is dissolved in 50 mL MiliQ water.
    ii. To dissolve EDTA, the pH needs to be 8.0. Therefore, NaOH is utilized to raise the pH. After dissolving EDTA, the pH is decreased to 7.0 by HCL.
2. Harvest peripheral blood.
  a. Use a sterile coupling system and a 20 mL syringe to inject 20 mL MACS buffer into the bag of G-CSF mobilized peripheral blood (approximately 30–50 mL).
  b. Massage the bag filled with buffer to detach and harvest all cells.
  c. Harvest the blood diluted with MACS buffer with the syringe.
  d. Detach the syringe from the coupling system while leaving the coupling system attached to the bag.
  e. Empty the syringe in a clean 50 mL tube.
  f. Repeat the procedure with another 20 mL MACS buffer.
  g. Count the cells in a Casy cell counter using 20 μL of the collected blood diluted with MACS buffer with 3 drops from a Pasteur pipette (approximately 20 to 50 μL per drop) of saponin to exclude red blood cells from the cell count.
  h. While counting the cells, spin the 50 mL tube containing the harvested blood for 5 min at 450 g with acceleration and brake 9 at RT.
  i. Remove the supernatant.
3. Isolate hematopoietic stem cells from G-CSF mobilized peripheral blood of healthy donors using CD34^+^ MACS beads according to the manufacturer’s protocol.
  a. Resuspend the cell pellet in MACS buffer (150 μL/100∗10^6^ PBMCs), Fc block (50 μL/100∗10^6^ PBMCs), and anti-CD34 beads ((50 μL/100∗10^6^ PBMCs).
    i. Add the Fc block before adding the anti-CD34 beads, no incubation needed.
  b. Mix and incubate for 30 min at 4°C.
  c. Wash the cells by adding 20 mL MACS buffer, spin for 5 min at 450 g with acceleration and brake 9 at RT.
  d. Remove the supernatant, detach the cells and add 500 μl MACS buffer per 100∗10^6^ PBMCs.
  e. Prepare the isolation magnet.
    i. Place an LS column in the magnet with a separation filter on top.
    ii. Place a waste tube below the column and separation filter.
    iii. Rinse the filter and column with 3 mL MACS buffer.
  f. Bring your cells onto the separation filter and wait until all volume has flowed through the column.
  g. Wash the column three times with 3 mL MACS buffer.
  h. Release the column from the magnet onto a clean 15 mL tube.
  i. Elute the cells with 5 mL MACS buffer.
  j. Spin cells for 5 min at 450 g with acceleration and brake 9 at RT.
  k. Repeat the isolation with an MS column by first setting up the column and magnet.
    i. Place an MS column in the magnet.
    ii. Place a waste tube below the column.
    iii. Rinse the column with 500 μL MACS buffer.
    iv. Resuspend the cells in 500 μL MACS buffer.
    v. Wash the column three times with 500 μL MACS buffer.
    vi. Elute the cells with 2 mL MACS buffer.
  l. Count the cells in the Casy using 20 μL of the 2 mL eluate (without saponin).
4. Check the purity of the isolated population using 30,000 cells for flow cytometry.
  a. Prepare 0.5% formaldehyde solution.
    i. Take 1622 μL formaldehyde and mix with 13.378 mL PBS for a 4% formaldehyde solution.
    ii. Take 5 mL of the 4% formaldehyde solution and add 35 mL PBS.
  b. Prepare FACS buffer.
    i. Take 500 mL sterile PBS and add 25 mL filter-sterile 10% BSA.
  c. Prepare CD34-APC labeling mix.
    i. Take 3 μL CD34-APC and add 27 μL FACS buffer.
  d. Label cells with the CD34-APC FACS buffer mix for 30 min at 4°C.
  e. Add 150 μL 0.5% formaldehyde for fixation of the cells.
5. Cryopreservation of CD34^+^ HSPCs.
  a. Prepare freeze/thaw medium.
    i. Take 500 mL sterile IMDM and add 100 mL FCS.
    ii. Place on ice.
  b. Prepare freezing medium with 10% DMSO.
    i. Take 900 μL freeze/thaw medium and add 100 μL DMSO in drops.
    ii. Place on ice.
  c. Spin the cells down for 5 min at 450 g with acceleration and brake 9 at 4°C.
  d. Remove the supernatant.
  e. Resuspend cells in 750 μL freeze/thaw medium and move your cells to a cryovial.
  f. Add 750 μL freezing medium with 10% DMSO in drops.
  g. Cells are frozen and stored in liquid nitrogen for preservation.

Similar results can be obtained with hematopoietic stem cells isolated from cord blood. However, due to the number of cells needed for bigger cell cultures, preference is given to CD34^+^ HSPCs from G-CSF mobilized peripheral blood donors. Another option would be to first expand CD34^+^ HSPCs from cord blood and then start the differentiation towards neutrophils.

##### Bringing CD34^+^ HSPCs in culture

6. Prepare freeze/thaw medium as described before (IMDM + 20% FCS).
7. Thaw CD34^+^ HSPCs by transferring the cells to a clean 50 mL tube and adding 20 mL ice-cold freeze/thaw medium in droplets.
8. Prepare complete culturing medium and store at 4°C.
a. Take either 500 mL of sterile IMDM or sterile SL-II.
b. Add 50 mL sterile FCS.
c. Add 5 mL 10^4^ U/mL penicillin/10 ng/mL streptomycin mix.
d. Add 5 mL 200 mM glutamine.
9. Prepare complete medium with growth factors.
  a. Depending on the number of cells that are thawed take per 1∗10^6^ cells.
    i. Take 2 mL complete medium (either IMDM or SL-II).
    ii. Add 2 μL 30 ng/mL G-CSF.
    iii. Add 10 μL 250 ng/mL Flt-3L.
    iv. Add 2 μL 10 ng/mL IL-3.
    v. Add 6 μL 10 ng/mL GM-CSF.
    vi. Add 5 μL 500 ng/mL hSCF.
  b. If the HSPCs do not replicate, ensure that you are using fresh growth factors. Especially human SCF is unstable and can be kept at 4 °C for one month maximum.
  c. Resuspend the cells in 2 mL of the complete medium containing growth factors per 1∗10^6^ cells to bring them to a concentration of 0.5∗10^6^ cells/mL.
  d. Transfer the cells to a polystyrene, non-pyrogenic cell culturing plate (for 1∗10^6^ cells use a 12 wells plate, for higher cell counts use a 6 wells plate).
  e. Leave cells in the incubator at 5% CO_2_ and 37°C.

##### Culturing CD34^+^ HSPCs towards neutrophils

10. Take complete medium out of the fridge and wait until it has reached room temperature (approximately 1 h).
11. Transfer the cells from the culturing dish to a clean 50 mL tube.
12. Wash the wells with 1 mL PBS and add to the cells in the 50 mL tube.
13. Count the cells by taking 20 μL of cell suspension into 10 mL isotonic solution in the Casy counter.
  a. If cell numbers are low after passing the cells, be careful with resuspending the cells in the plates before transferring the cells and make sure that you rinse the wells with PBS.
14. Prepare complete medium with G-CSF depending on the cell number.
  a. Per 1∗10^6^ cells take 2 mL of complete medium.
  b. Add 2 μL 10 ng/mL G-CSF.
  c. Invert the tube to mix well.
15. Spin down cells for 5 min at 300 g for 5 min with acceleration and brake 9 at RT.
16. Resuspend cells in complete medium supplemented with G-CSF and transfer to a new culturing plate or culturing flask depending on the volume.
17. After day 7, resuspend cells at 1∗10^6^ cells/mL instead of 0.5∗10^6^ cells/mL.

HSPCs and neutrophils are very sensitive. If cells are pre-activated they start to clump together, releasing their DNA and becoming sticky. Try to avoid this by resuspending cells gently and also spin down slowly (maximum 300 g). Also ensure gentle closure of incubator doors while culturing cells.

### Quantification and statistical analysis

For statistical analysis we used Graphpad Prism version 10.3 (GraphPad software, Boston, Massachusetts, USA). P values lower than 0.05 were considered statistically significant and were indicated as ∗ *p* <0.05, ∗∗ *p* <0.01, ∗∗∗ *p* <0.001 and ∗∗∗∗ *p* <0.0001. Statistical details of each experiment can be found in the corresponding figure legends. n represents the number of individual donors tested.

#### Institutional permissions

Approval from your local ethics committee is required to isolate and culture cells from healthy donors. Furthermore, approval of these donors is required. The donors from which G-CSF mobilized peripheral blood was obtained were enrolled in a coded system for anonymous use of blood products in accordance with Dutch regulations following the declaration of Helsinki. Informed consent was collected from the donors and approved by the Sanquin Internal Ethical Advisory Board (Privacy Declaration 1909–0176).
